# Evidence for a Physiological Role of T-Type Ca Channels in Ventricular Cardiomyocytes of Adult Mice

**DOI:** 10.3390/membranes12060566

**Published:** 2022-05-28

**Authors:** Jessica Marksteiner, Janine Ebner, Isabella Salzer, Elena Lilliu, Benjamin Hackl, Hannes Todt, Helmut Kubista, Seth Hallström, Xaver Koenig, Karlheinz Hilber

**Affiliations:** 1 Department of Neurophysiology and Pharmacology, Center for Physiology and Pharmacology, Medical University of Vienna, 1090 Vienna, Austria; jessica.marksteiner@meduniwien.ac.at (J.M.); janine.ebner@meduniwien.ac.at (J.E.); isabella.salzer@meduniwien.ac.at (I.S.); elena.lilliu@meduniwien.ac.at (E.L.); benjamin.hackl@meduniwien.ac.at (B.H.); hannes.todt@meduniwien.ac.at (H.T.); helmut.kubista@meduniwien.ac.at (H.K.); 2 Division of Physiological Chemistry, Otto Loewi Research Center, Medical University of Graz, 8036 Graz, Austria; seth.hallstroem@medunigraz.at; 3 Ludwig Boltzmann Institute for Cardiovascular Research at the Center for Biomedical Research, Medical University of Vienna, 1090 Vienna, Austria

**Keywords:** adult murine heart, intracellular Ca release, T-type Ca channels, whole cell patch clamp, working myocardium

## Abstract

T-type Ca channels are strongly expressed and important in the developing heart. In the adult heart, these channels play a significant role in pacemaker tissues, but there is uncertainty about their presence and physiological relevance in the working myocardium. Here, we show that the T-type Ca channel isoforms Cav3.1 and Cav3.2 are expressed at a protein level in ventricular cardiomyocytes from healthy adult C57/BL6 mice. Myocytes isolated from adult wild-type and Cav3.2 KO mice showed considerable whole cell T-type Ca currents under beta-adrenergic stimulation with isoprenaline. We further show that the detectability of basal T-type Ca currents in murine wild-type cardiomyocytes depends on the applied experimental conditions. Together, these findings reveal the presence of functional T-type Ca channels in the membrane of ventricular myocytes. In addition, electrically evoked Ca release from the sarcoplasmic reticulum was significantly impaired in Cav3.2 KO compared to wild-type cardiomyocytes. Our work implies a physiological role of T-type Ca channels in the healthy adult murine ventricular working myocardium.

## 1. Introduction

Two low-voltage-activated T-type Ca channel isoforms, namely Cav3.1 and Cav3.2, are expressed in the mammalian heart. Both isoforms are prevalent in the developing heart, but downregulated in the myocardium shortly after birth [1]. In healthy adult hearts, T-type Ca channels are mainly localized to the sinus node, the atrioventricular node, and to the Purkinje fibers of the ventricular conduction system, where they have an established role in pacemaker function [2,3]. In cardiomyocytes of the adult ventricular working myocardium, functional T-type Ca channel expression is most commonly considered to be low or even missing. However, these channels are re-expressed in cardiovascular disease states and after injury [1,3,4,5,6].

Several findings from previous studies have challenged the view that T-type Ca channels are irrelevant in the healthy adult ventricular cardiomyocytes of mammals. Among those findings is the existence of a considerable T-type Ca current (ICaT) in ventricular myocytes isolated from the hearts of adult animals of various species (for review see [5]) including mice [7,8], hamsters [9], and guinea pigs [10,11]. In guinea pig ventricular myocytes, T-type Ca channels have even been shown to contribute to excitation-contraction (EC)-coupling [11]. On the other hand, numerous other studies have failed to detect significant ICaTs in ventricular myocytes isolated from adult mammals (e.g., [12,13,14,15]). The reason for this apparent inconsistency is unclear, but may relate to differences in the employed methodological approaches. For example, variations in myocyte dissociation procedures, cell conservation methods, patch clamp pulse protocols, and the composition of experimental solutions may have contributed to overlooking actually present ICaT.

In an attempt to shed more light on this inconsistency, we reinvestigated selected issues of T-type Ca channel expression and function in ventricular cardiomyocytes isolated from adult mouse hearts. Against the prevailing doctrine, we provide evidence for a physiological role of T-type Ca channels in the healthy adult murine ventricular myocardium.

## 2. Materials and Methods

### 2.1. Ethical Approval

The investigation coincides with the guiding principles of the Declaration of Helsinki and conforms to the rules of our University Animal Welfare Committee. The ethics vote for keeping and breeding mice for organ withdrawal from sacrificed animals has the following number: BMWFW-66.009/0175-WF/V/3b/2015.

### 2.2. Mice

Cacna1h (Cav3.2) knock-out mice (strain B6;129-Cacna1htm1Kcam/J; stock No: 013770) and respective wild-type (wt) control mice (C57BL/6J; stock No: 000664) were purchased from the Jackson Laboratory. C57BL/10J mice originated from the Charles River Laboratories.

### 2.3. Isolation of Ventricular Cardiomyocytes

Cav3.2 KO (4 females, 4 males), wt BL/6 (4 females, 4 males), and wt BL/10 (1 female, 4 males) mice in an age range between 15 and 20 weeks were anaesthetized using isoflurane (2%, inhalation) and euthanized by cervical dislocation. Thereafter, cardiomyocytes were isolated from the ventricles of their hearts using a Langendorff setup according to the myocyte isolation procedure described in our previous study [16].

### 2.4. Detection of Ca Currents

Ca currents were recorded in the whole-cell mode of the patch-clamp technique from cardiomyocytes up to 8 h after preparation at an experimental temperature of 22 ± 1.5 °C using an Axoclamp 200 B patch-clamp amplifier (Axon Instruments, Union City, CA, USA). Pipettes were formed from aluminosilicate glass (AF150—100–10, Science Products, Hofheim, Germany) with a P-97 horizontal puller (Sutter Instruments, Novato, CA, USA), and had resistances between 1 MΩ and 1.8 MΩ when filled with internal solution (see below). Data acquisition was performed with pClamp 11.0 software (Axon Instruments, Union City, CA, USA) through a 16-bit A-D/D-A interface (Digidata 1440; Axon Instruments, Union City, CA, USA). Data were low-pass filtered with 2 kHz (3 dB) and digitized at 5 kHz. Leak currents and capacity transients were subtracted by using a P/4 protocol. Data were analyzed with Clampfit 10.7 (Axon Instruments) and Prism 5.04 (GraphPad Software, San Diego, CA, USA) software. Bath solutions were exchanged via a DAD-8-VC superfusion system (ALA Scientific Instruments, Westbury, NY, USA). The compositions of the recording solutions (standard and solution set 2 [17]) are given in Table 1. T-type and L-type Ca currents were normally elicited from a holding potential of −95 mV by depolarizing voltage steps up to +50 mV. In some experiments, a holding potential of −135 mV was used. Membrane voltages were corrected for liquid junction potentials. For the generation of current density–voltage relations, the current amplitudes at various voltages were measured and then divided by the cell capacitance to derive the current density values. Then, the values were plotted against the respective test pulse voltages. To force the occurrence of currents through T-type Ca channels (hardly present under basal conditions), cardiomyocytes were superfused with bath solution containing 100 nM of the β-receptor agonist isoprenaline.

### 2.5. Immunostaining

Isolated ventricular cardiomyocytes were plated on cover slips and, after 90 min, fixed in 4% paraformaldehyde for 10 min at room temperature. Thereafter, the culture medium was removed and the myocytes were washed three times with PBS, permeabilized in 0.1% Triton X-100 (Sigma-Aldrich, Vienna, Austria) for 5 min at room temperature, and washed again three times with PBS. This was followed by blocking with 10% goat serum (Sigma-Aldrich, Vienna, Austria) and 0.01% azide (Sigma-Aldrich, Vienna, Austria) in PBS for 2 h. Subsequently, the cells were incubated with a selective anti-Cav3.1 antibody (anti-CACNA1G, #ACC-021, source: rabbit, Alomone Labs, Jerusalem, Israel, 1:500) or with one of two selective anti-Cav3.2 antibodies (anti-CACNA1H, #ACC-025, source: rabbit, Alomone Labs, Jerusalem, Israel, 1:1000; anti-Cav3.2 polyclonal antibody, #PA5-106771, source: rabbit, Invitrogen, Paisley, England, 1:200) at 4 °C overnight. The following day, cells were rinsed three times with PBS and incubated with the secondary antibody (Alexa Fluor 488, #A11008, goat anti-rabbit, Invitrogen, Paisley, England, 1:500) for 60 min at room temperature. After three subsequent PBS washing steps, the myocytes were mounted, dried, and stored at 4 °C. The slides were finally analyzed using an LSM 510 confocal microscope (Zeiss, Jena, Germany). For the immunostaining experiments, 3 wt BL/6 mice (15–20 weeks of age) were used for cell isolation. The microscope settings were always set so that there was no fluorescence signal when omitting the respective primary antibody in an experiment. This excluded signals due to the unspecific binding of the secondary antibody.

### 2.6. Intracellular Ca Measurements

Ca transients were recorded from isolated wt and Cav3.2 KO mouse ventricular cardiomyocytes in 3.5 cm culture dishes at room temperature following the protocol described in our previous study [18]. In brief, myocytes pre-loaded with the cell membrane-permeable Ca-sensitive fluorescent dye Fluo-4 AM (Thermo Fisher Scientific, Vienna, Austria) were bathed in an external solution containing (in mmol/L) 140 NaCl, 4 KCl, 2 CaCl_2_, 2 MgCl_2_, 5 HEPES, and 5 glucose, with pH adjusted to 7.4 with NaOH. Electrical stimulation via platinum electrodes in the bath was performed at 0.1 Hz in order to elicit Ca transients. To elicit caffeine-induced Ca release, bath solution containing 20 mmol/L caffeine was applied via an OctaFlow II perfusion system (ALA Scientific Instruments, Westbury, NY, USA). Culture dishes were mounted on a confocal microscope (Nikon A1R+, Düsseldorf, Germany) equipped with a 12 kHz resonant scanner. Fluo-4 was excited at 488 nm and dye- emitted fluorescence signals were collected at 525/50 nm. Then, xyt image series were obtained with a sample rate of 17 ms (60 frames per sec). The use of xyt instead of line scans allowed the simultaneous imaging of several cardiomyocytes within a field of view while allowing a sampling fast enough for the adequate recording of Ca transient amplitudes and decay kinetics. A change in fluorescence (F) peaks upon stimulation with single electrical pulses or with caffeine was evaluated relative to baseline fluorescence (F0) prior to stimulation and is given as F/F0 throughout the manuscript. To evaluate the duration of the elicited Ca transients, a single exponential function was fitted to the decaying fluorescence to obtain respective time constants (ԏ-values).

### 2.7. Statistical Analyses

Data are expressed as means ± SEM. Statistical comparisons between cardiomyocytes before and during isoprenaline application after the steady-state was reached were made using a paired two-tailed Student’s *t*-test. The same test was used for comparisons between experiments at −95 mV and −135 mV holding potential. Comparisons between wt and Cav3.2 KO myocytes were carried out with an unpaired two-tailed Student’s *t*-test. *p* < 0.05 was considered significant.

## 3. Results

**Figure 1:**
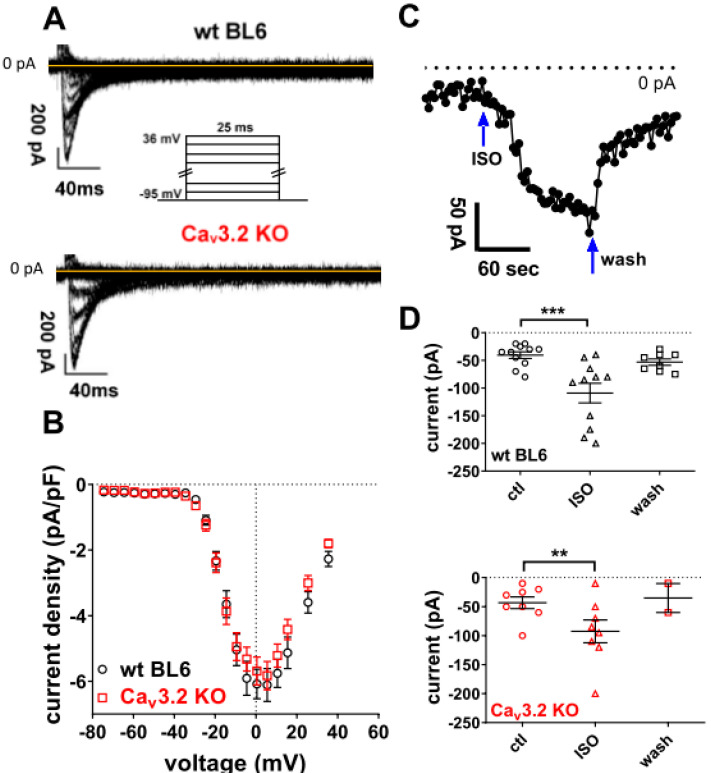
Ca currents in wt and Cav3.2 KO ventricular cardiomyocytes. (**A**) Original traces of Ca currents of a typical wt and Cav3.2 KO myocyte elicited by the pulse protocol displayed on top. (**B**) Current density–voltage relationships of wt (n = 20) and Cav3.2 KO (n = 20) myocytes. Data are expressed as means ± SEM. The current density–voltage relations hardly revealed any T-type Ca channel activity (expected to be maximal at −45 mV) but did reveal significant L-type Ca channel activity (peaking at +5 mV), which was similar in wt and Cav3.2 KO myocytes (*p* = 0.67 at +5 mV, unpaired Student’s *t*-test). Capacitance values of the examined myocytes were 184 ± 13 pF for wt and 169 ± 11 pF for Cav3.2 KO (*p* = 0.37). (**C**) The effect of the external application of 100 nM isoprenaline (ISO) on ICaT in a wt myocyte. From a holding potential of −95 mV, depolarizing voltage steps to −45 mV were applied every 3 s to elicit the currents. The arrows indicate the beginning and end of ISO application. Current peaks were finally plotted against time. (**D**) ICaT peaks before, during application after the steady-state was reached, and after the washout of ISO in wt (top) and Cav3.2 KO (bottom) myocytes. There was no significant difference between wt and Cav3.2 KO under control conditions (*p* = 0.81, unpaired Student’s *t*-test) or in the presence of ISO (*p* = 0.54). Data are expressed as means ± SEM. Each data point represents a single cell. ** *p* < 0.01; *** *p* < 0.001; paired Student’s *t*-test.

We first investigated the presence of ICaT in ventricular cardiomyocytes isolated from adult wild-type (wt) C57/BL6 and Cav3.2 KO mice. Figure 1A,B shows that neither wt, nor Cav3.2 KO myocytes exhibited significant basal ICaT, whereas a considerable L-type Ca current (ICaL) was observed. When related to the respective L-type current amplitudes (100%), T-type current amplitudes amounted to less than 5% both in wt and Cav3.2 KO myocytes. In order to test if beta-adrenergic stimulation of the myocytes would enable the triggering of considerable ICaT by our pulse protocol (inefficient at basal conditions), we superfused the cells with 100 nM isoprenaline. The activation of ICaT in cardiac myocytes by isoprenaline has previously been reported [5,19]. Figure 1C,D shows that isoprenaline application significantly increased ICaT in a reversible manner. This suggests the presence of functional T-type Ca channels responsive to beta-adrenergic stimulation in the adult cardiomyocyte membrane. Both wt and Cav3.2 KO myocytes responded to isoprenaline and the current enhancement generated by the drug was only somewhat greater in wt cells (Figure 1D). This implies that a major part of the isoprenaline-induced ICaT was carried by Cav3.1 channels.

**Figure 2:**
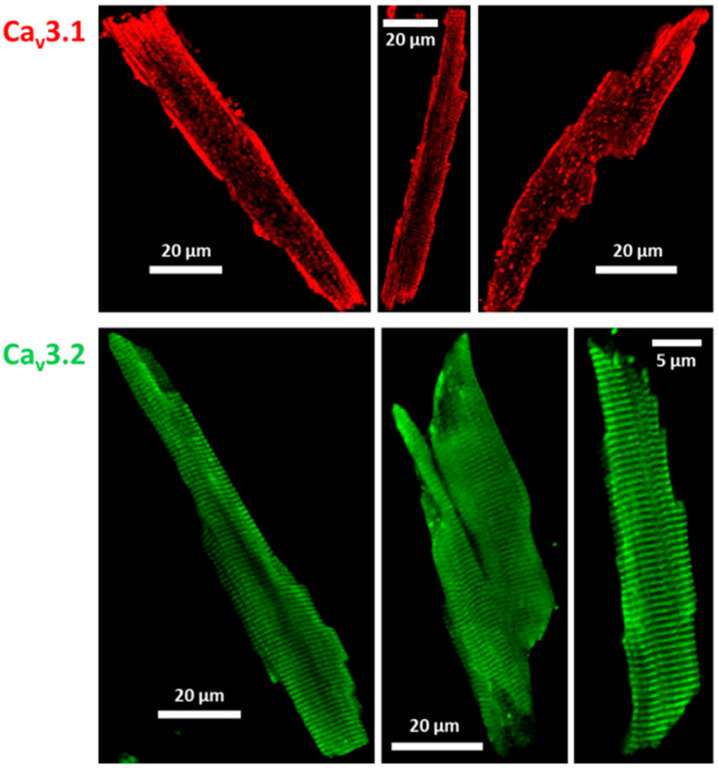
Immunostaining of T-type Ca channels in ventricular cardiomyocytes isolated from adult wt mice. Cav3.1 (**top**) channel expression and localization was detected using a selective anti-Cav3.1 antibody (anti-CACNA1G, #ACC-021, source: rabbit, Alomone Labs, 1:500). Cav3.2 (**bottom**) was detected with the selective anti-Cav3.2 antibodies (anti-CACNA1H, #ACC-025, source: rabbit, Alomone Labs, 1:1000; left and right cell; or anti-Cav3.2 polyclonal, #PA5-106771, source: rabbit, Invitrogen, 1:200; middle cell). Secondary antibody: Alexa Fluor 488, #A11008, goat anti-rabbit, Invitrogen, 1:500.

Next, we intended to confirm the presence of T-type Ca channels in ventricular cardiomyocytes from adult wt BL/6 mice at the protein level. We performed immunostaining experiments on isolated myocytes, using specific antibodies for Cav3.1 and Cav3.2. Figure 2 shows that both Cav3.1 and Cav3.2 channels were expressed. Cross-striations typical for T-tubular localization in ventricular cardiomyocytes were observed with both antibodies directed against Cav3.2, but not with the antibody directed against Cav3.1. These results suggest a different cellular localization of Cav3.1 and Cav3.2 whereby only the latter channel isoform appears to be present in the T-tubular system.

**Figure 3:**
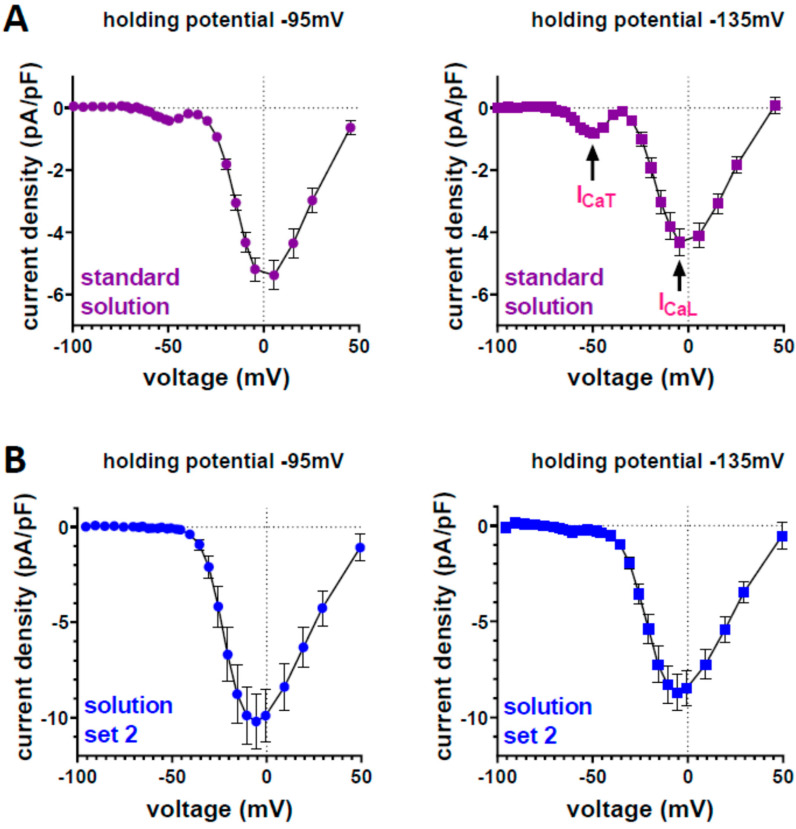
Ca current density–voltage relationships recorded from ventricular cardiomyocytes isolated from wt BL/10 mouse hearts under different experimental conditions. (**A**) Left: standard solution (see Materials and Methods) at holding potential −95 mV; T/L-type current amplitude: 9%; Right: standard solution at holding potential −135 mV; T/L amplitude: 19%; a significant difference existed between different ICaT amplitudes at −50 mV (*p* = 0.006, paired Student’s *t*-test). Data represent means ± SEM (n = 11). (**B**) Left: solution set 2 (see Materials and Methods) at holding potential: −95 mV; T/L amplitude: 0%; Right: solution set 2 at holding potential: −135 mV; T/L amplitude: 5% (n = 14).

The significant expression of T-type Ca channels but a lack of basal ICaT in ventricular cardiomyocytes suggested that our experimental conditions were possibly inappropriate for ICaT detection. We therefore varied experimental specifics to make ICaT detectable. For these experiments we used C57/BL10 wt mice, since we have previously observed basal ICaT in myocytes from adult BL/10 [7,8], but not BL/6 ([20]; current study) mice. Using identical experimental solutions and procedures as in the studies described above, we found a marginal ICaT in BL/10 wt myocytes (Figure 3A). This current was significantly increased when the holding potential at the recordings was changed from −95 mV to −135 mV. In another set of experiments, we used solution set 2 instead of the standard experimental solutions (see Section 2: Materials and Methods) for ICa recordings. Under these conditions, considerable ICaT was lacking, independent of the applied holding potential (Figure 3B). These findings reveal that the presence and/or size of ICaT in murine wt myocytes is dependent on the applied experimental conditions.

**Figure 4:**
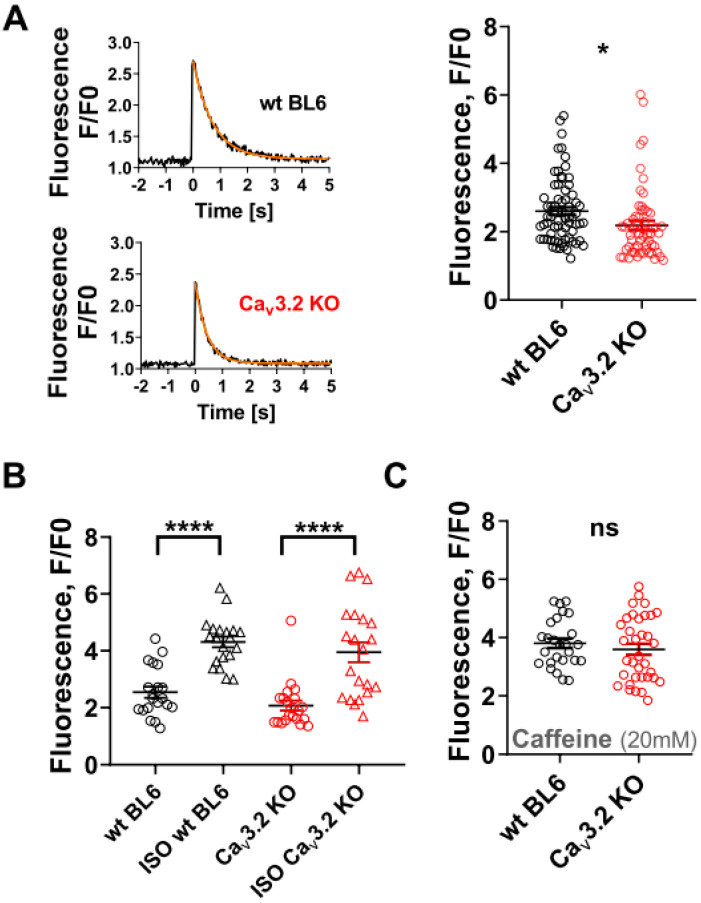
Electrically induced and caffeine-induced intracellular Ca transients in wt and Cav3.2 KO ventricular cardiomyocytes. (**A**) Left: Representative time courses of Fluo-4 fluorescence reporting rises in cytosolic Ca concentration during electrical field stimulation at 0.1 Hz frequency in a single wt and Cav3.2 KO myocyte. The orange lines represent fits of the data with a single exponential function. Right: Comparison of mean Ca peak fluorescence relative to baseline (F/F0) between wt and Cav3.2 KO myocytes. Each data point represents a single cell, and values are expressed as means ± SEM (n= 68 for wt and 59 for Cav3.2 KO myocytes). * *p* < 0.05 (0.02); unpaired Student’s *t*-test. The Ca transients decayed with time constants of 0.30 ± 0.02 s in wt and 0.25 ± 0.01 s in Cav3.2 KO myocytes, which were significantly different (*p* = 0.04). (**B**) Comparison of mean Ca peak fluorescence (F/F0) before and after the application of 100 nM ISO in wt (n = 20) and Cav3.2 KO (n = 21) myocytes. **** *p* < 0.0001 (paired Student’s *t*-test). There was no significant difference between wt and Cav3.2 KO in the presence of ISO (*p* = 0.37; unpaired Student’s *t*-test). The Ca transients under ISO decayed with time constants of 0.14 ± 0.02 s in wt and 0.11 ± 0.02 s in Cav3.2 KO myocytes (not significantly different, *p* = 0.19). (**C**) Comparison of mean Ca peak fluorescence elicited by an application of 20 mM caffeine, relative to baseline (F/F0), between wt (n = 26) and Cav3.2 KO (n = 35) myocytes. ns—not significant (unpaired Student’s *t*-test, *p* = 0.42).

During the plateau phase of the cardiac action potential, the Ca influx through Ca channels into the cell triggers Ca-induced Ca release from the sarcoplasmic reticulum (SR), which finally initiates contraction. To check whether the presence of Cav3.2 channels affected Ca release, we compared electrically evoked intracellular Ca transients between wt and Cav3.2 KO myocytes. Figure 4A shows that the amplitude of the Ca transients was significantly decreased in Cav3.2 KO myocytes by 16%. This suggests an abnormally reduced Ca release in the absence of Cav3.2. In addition, the decay of the Ca transient was significantly quicker in Cav3.2 KO, compared to wt myocytes (see legend of Figure 4). This indicates a quicker removal of Ca from the cytosol after SR Ca release in the absence of Cav3.2. However, this latter effect was only moderate and on the border of reaching statistical significance (*p* = 0.04), and is therefore not further interpreted in the discussion section. Figure 4B shows the effect of 100 nM isoprenaline on Ca transient peaks in wt and Cav3.2 KO myocytes. It can be observed that the drug generated similar increases in Ca transient amplitudes, revealing that the responsiveness to beta-adrenergic stimulation was not impaired in the absence of Cav3.2. In a final set of experiments, we used the ryanodine receptor agonist caffeine to elicit cytosolic Ca transients (Figure 4C). The caffeine-induced Ca transient amplitude is a measure for SR Ca load [21]. We observed that Ca transients elicited by caffeine have similar amplitudes in wt and Cav3.2 KO myocytes.

## 4. Discussion

The functional relevance of T-type Ca channels in the adult mammalian heart is well established and commonly ascribed to pacemaker tissue [2,3] Accordingly, Cav3.1 KO mice have abnormally decreased heart rates and slowed atrioventricular conduction [22,23]. T-type Ca channels are also involved in the remodeling of the adult heart [1,3,5], e.g., Cav3.2 induces cardiac hypertrophy [23,24] and T-type Ca channel blockade has been shown to be protective in mice with heart failure [20]. A comprehensive description of the physiological roles of T-type Ca channels in the heart can be found in Vassort et al. [5].

### 4.1. Physiological Role of T-Type Ca Channels in Ventricular Cardiomyocytes of Healthy Adult Mice

In the present study, we report evidence for a physiological role of T-type Ca channels in the healthy adult ventricular working myocardium unrelated to cardiac pacemaker function. The evidence includes: (1) the presence of small basal ICaTs under appropriate experimental conditions in ventricular cardiomyocytes isolated from healthy adult mice; (2) the presence of ICaTs induced by beta-adrenergic stimulation; (3) Cav3.1 and Cav3.2 channel expression at the protein level in myocytes; and (4) significantly reduced electrically evoked intracellular Ca transient amplitudes in Cav3.2 KO compared to wt myocytes. We conclude from the presence of Cav3.x channel protein and ICaTs that a certain fraction of T-type Ca channels is functionally expressed in the adult murine ventricular working myocardium. In terms of physiological membrane potential, however, the ICaT may be quite small, which narrows its potential functional relevance. Reduced Ca transient amplitudes in Cav3.2 KO cells suggest that a lack of normally present Cav3.2 channels in ventricular cardiomyocytes impairs Ca release from the SR by ryanodine receptors (RYRs). This is in line with Sipido et al., reporting a contribution of T-type Ca channels to excitation–contraction coupling in ventricular myocytes isolated from adult guinea pigs [11]. Smaller Ca transients in Cav3.2 KO compared to wt ventricular myocytes are also in agreement with increased Ca transients and contractility in myocytes derived from a mouse line with the cardiac-specific over-expression of T-type Ca channels [25]. Interestingly, these authors further report that SR Ca load was not affected by the over-expression of T-type Ca channels. Together with our finding of a normal SR Ca load in Cav3.2 KO myocytes (Figure 4C), this implicates that the loading of the SR with Ca is independent of T-type Ca channel expression. Finally, smaller Ca transients in Cav3.2 KO compared to wt ventricular myocytes are also in line with decreased Ca transient peaks due to the blocking of ICaT observed using a mathematical model of mouse atrial myocytes [26].

Based on our data, the impairment of electrically evoked Ca release in Cav3.2 KO compared to wt myocytes cannot be explained by diminished Ca release-triggering ICaTs. Thus, neither Cav3.2 KO, nor wt BL/6 myocytes exhibited considerable ICaT under basal conditions (Figure 1B). In addition, most of the whole cell ICaT in the presence of isoprenaline seems to be carried by Cav3.1 and not Cav3.2 channels (Figure 1D). It is, however, possible that the presence of Cav3.2 channels in wt myocytes enables local Ca signaling (undetectable in our system) through interaction with RYRs in close proximity, resulting in enhanced total Ca release from the SR. This is in line with the expression of Cav3.2, but not Cav3.1 channels, in T-tubules (Figure 2) in the vicinity of the RYR harboring SR membrane. Moreover, in murine vascular smooth muscle cells, Cav3.2 channels are expressed in the caveolae and positioned close to RYRs to allow extracellular Ca influx to trigger Ca sparks [27,28]. The caveolar localization of Cav3.2, suggesting a similarly tight coupling of these channels with RYRs, has also been reported for neonatal and diseased adult ventricular cardiomyocytes from mice [29,30]. If also true for healthy adult ventricular cardiomyocytes, the loss of coupling between Cav3.2 in the caveolae (and/or T-tubules) and RYRs would explain the decreased Ca release in Cav3.2 KO myocytes. The precise mechanism of Cav3.2-induced Ca transient modulation remains unknown. Since Cav3.2 is important in development and differentiation of ventricular myocytes, we also want to mention the possibility that Cav3.2 KO may alter the expression levels of EC-coupling proteins such as RyR or their cluster structure.

Taken together, functional T-type Ca channels are expressed in ventricular cardiomyocytes of the working myocardium of healthy adult mice, and the lack of Cav3.2 channels impairs cellular Ca handling. Besides the established role that T-type Ca channels play in the adult murine heart with regard to pacemaker function, their activity in ventricular myocytes may additionally modulate excitation–contraction coupling and contractility. L-type Ca channel activity, however, remains the main player in the working myocardium.

### 4.2. Potential Factors Responsible for Overlooking Actually Present ICaT in the Ventricular Cardiomyocytes of Adult Mammals

Similar to numerous previous studies on ventricular cardiomyocytes isolated from the hearts of adult mammalian species (e.g., [12,13,14,15]), we did not detect significant ICaT in myocytes from adult BL/6 mice under basal conditions. Considerable ICaT, however, occurred during beta-adrenergic stimulation using isoprenaline. Obviously, T-type Ca channels resided in the membrane, but were somehow “silenced” as they could not be considerably activated by our voltage-clamp protocol in the absence of beta-adrenergic stimulation. This phenomenon may also have occurred in studies by other authors.

Potential reasons for silenced T-type Ca channels or the lack of basal ICaT in isolated myocytes include aggressive enzymatic treatments for cell dissociation, the rapid down-regulation of channels after cell isolation, intracellular signaling molecule loss through the pipette tip during ICaT recordings with the whole cell patch clamp technique, and difficulties in separating small ICaTs from large, partly overlaying ICaLs. The latter issue especially applies if barium instead of Ca is used as a charge carrier (see our previous studies [7,8]). In the present study, we experimentally tested the previously suggested view that holding potentials near the resting membrane potential may reduce T-type Ca channel availability [31,32]. Indeed, we found a significantly increased ICaT at −135 compared with −95 mV holding potential. As ICaT recordings near the membrane resting potential (i.e., −80 mV holding potential) are common practice, it is not surprising that considerable ICaT has rarely been detected. Moreover, our experiments have also shown that the presence of ICaT critically depends on the composition of the experimental solutions (Figure 3). The use of the standard solutions, but not solution set 2 (see Materials and Methods), generated considerable ICaT. The most striking difference between the two solution sets was the concentration of EGTA in the pipette solution (0.1 versus 10 mM, respectively). However, we can, at present, only speculate that high internal EGTA concentrations may impair ICaT detectability in the recordings. Finally, as already mentioned in the results, when using murine ventricular cardiomyocytes, the genetic background of the mice seems to play a role. In our studies, considerable basal ICaT was observed in myocytes from adult BL/10 ([7,8]; current study), but not BL/6 ([20]; current study) mice.

Based on our results, we speculate that the presence of ICaT in ventricular cardiomyocytes from adult mammals is often overlooked or at least underestimated due to inadequate methodological approaches. As already suggested more than 15 years ago [5], it would be exciting to clarify the real characteristics and role of ICaT in the ventricular myocardium studied on multicellular preparations under suitable voltage-clamp conditions, superfused with physiological solutions, not treated by enzymes, and not intracellularly dialyzed.

## Figures and Tables

**Table 1:** Composition of the recording solutions.

Standard Solution		Solution Set 2	
Bath solution (mM)	Pipette solution (mM)	Bath solution (mM)	Pipette solution (mM)
TEA-Cl (145)	Cs-aspartate (145)	NMDG (150)	CsCl (102)
HEPES (10)	HEPES (10)	HEPES (15)	HEPES (10)
CaCl_2_ (2)	MgCl_2_ (2)	Glucose (5)	EGTA (10)
TEA-OH to pH 7.4	Mg-ATP (2)	CsCl (5)	TEA-Cl (10)
	Cs-EGTA (0.1)	CaCl_2_ (2)	MgCl_2_ (5)
	CsOH to pH 7.4	HCl to pH 7.4	Na_2_ATP (5)
			pH 7.4

## Data Availability

The data presented in this study are available on request from the corresponding author.
